# Renin-Angiotensin-Aldosterone System Inhibitors and Risk of Cancer: A Population-Based Cohort Study Using a Common Data Model

**DOI:** 10.3390/diagnostics12020263

**Published:** 2022-01-21

**Authors:** Seung-Hwa Lee, Jungchan Park, Rae Woong Park, Seo Jeong Shin, Jinseob Kim, Ji Dong Sung, Dae Jung Kim, Kwangmo Yang

**Affiliations:** 1 Rehabilitation and Prevention Center, Heart Vascular Stroke Institute, Samsung Medical Center, Sungkyunkwan University School of Medicine, Seoul 06351, Korea; shuaaa.lee@samsung.com (S.-H.L.); jidong.sung@samsung.com (J.D.S.); 2 Department of Biomedical Engineering, Seoul National University College of Medicine, Seoul 03080, Korea; 3 Department of Anesthesiology and Pain Medicine, Samsung Medical Center, Sungkyunkwan University School of Medicine, Seoul 06351, Korea; j83.park@samsung.com; 4 Department of Biomedical Sciences, Ajou University Graduate School of Medicine, Suwon 16499, Korea; rwpark99@gmail.com (R.W.P.); lucid900921@naver.com (S.J.S.); 5 Department of Epidemiology, School of Public Health, Seoul National University, Seoul 03080, Korea; jinseob2kim@gmail.com; 6 Department of Endocrinology and Metabolism, Ajou University School of Medicine, Suwon 16499, Korea; djkim@ajou.ac.kr; 7 Center for Health Promotion, Samsung Medical Center, Sungkyunkwan University School of Medicine, Seoul 06351, Korea

**Keywords:** renin-angiotensin-aldosterone system inhibitors, cancer occurrence

## Abstract

Studies have reported conflicting results on the association between the use of renin-angiotensin-aldosterone system (RAAS) inhibitors and cancer development. We compared the incidence of cancer between patients using RAAS inhibitors and other antihypertensive drugs. This retrospective observational cohort study used data from seven hospitals in Korea that were converted for use in the Observational Medical Outcomes Partnership Common Data Model. A total of 166,071 patients on antihypertensive therapy across the databases of the seven hospitals were divided into two groups according to the use of RAAS inhibitors. The primary outcome was the occurrence of cancer. A total of 166,071 patients across the databases of the seven hospitals was included in the final analysis; 26,650 (16%) were in the RAAS inhibitors group and 139,421 (84%) in the other antihypertensive drugs group. The meta-analysis of the whole cohort showed a lower incidence of cancer occurrence in the RAAS inhibitor group (9.90 vs. 13.28 per 1000 person years; HR, 0.81; 95% confidence interval [CI], 0.75–0.88). After propensity-score matching, the RAAS inhibitor group consistently showed a lower incidence of cancer (9.90 vs. 13.28 per 1000 person years; HR, 0.86; 95% CI, 0.81–0.91). The patients using RAAS inhibitors showed a lower incidence of cancer compared with those using other antihypertensive drugs. These findings support the association between the use of RAAS inhibitors and cancer occurrence.

## 1. Introduction

There have been conflicting results about the association between renin-angiotensin-aldosterone system (RAAS) inhibitor and cancer. The use of RAAS inhibitors has been associated with cancer development [1,2,3,4]. The majority of studies have reported that the use of RAAS inhibitors was significantly associated with a lower risk of cancer, but some studies failed to demonstrate a clinical significance [5] or found that RAAS inhibitors can increase cancer occurrence [1]. RAAS could affect cancer development or prevention by several mechanisms, such as tumor angiogenesis, cell survival and proliferation, immunomodulation, and tumor-induced fibrosis [6]. Relevant factors such as types of RAAS inhibitors, contaminants, various receptors, polymorphism, and cancer subtypes have been suggested to explain these discrepancies, but the results have not been conclusive [4,6]. For example, high levels of angiotensin II type 1 receptor (AT1R), which regulates Angiotensin II, are found in breast hyperplasia but decrease in invasive breast cancer while in the ovarian carcinoma up-regulation of AT1R associated with tumor invasiveness. Hence, an effect of RAAS inhibitors on cancer development has not been concluded.

Recently, using real-world data with a large number of patients has drawn attention to add evidence to such a matter of large debate. In this study, we conducted an analysis of patients from seven hospitals using the cohort generated by the Observational Medical Outcomes Partnership Common Data Model (OMOP-CDM) [7], which is a widely used method for converting heterogeneous data into a form that uses standardized vocabularies, so that international researchers can apply the same study protocol and codes to data from other regions of the world. Using this data, we enrolled patients who were diagnosed with hypertension and were prescribed antihypertensive medication. We compared the incidence of cancer between patients using RAAS inhibitors and those using other antihypertensive drugs, and the results are also presented by cancer type.

## 2. Materials and Methods

The Institutional Review Board at Ajou University Hospital waived the approval and the requirement for informed consent for this study (MED-MDB-21-125), considering that the data for this study were de-identified and based on longitudinal observational health data from the Observational Health Data Sciences and Informatics (OHDSI) research network. This retrospective observational cohort study was conducted in accordance with the principles of the Declaration of Helsinki and reported according to the Strengthening the Reporting of Observational Studies in Epidemiology statement [7].

### 2.1. Data Curation, Cohort Definition, and Outcome

OHDSI is a worldwide non-profit research alliance that provides open-source analysis for medical big data. The OHDSI CDM provides standard-based data analysis solutions that support converting electronic health record (EHR) data from different sources into a standard data structure, representing EHR data using semantically consistent concepts and conducting large-scale data analysis. A large-scale multicenter survival analysis could be conducted using the OHDSI platform. The data from the seven hospitals (Ajou University Medical Center, Daegu Catholic University Medical Center, Kangwon National University Hospital, Kangdong Sacred Heart Hospital, Pusan National University Hospital, Kyung Hee University Hospital at Gangdong, and Wonkwang University Hospital) in Korea were mapped to the OMOP-CDM for this analysis. The target cohort was generated by selecting adult patients prescribed RAAS inhibitors, and the comparator cohort was generated by selecting the remaining patients who used other antihypertensive drugs. Patients prescribed an RAAS inhibitor for less than one year, those with a prescription of both RAAS inhibitor and other types of antihypertensive drugs, or those with a cancer diagnosis within one year before the prescription date of antihypertensive drugs were excluded. In the OMOP-CDM, baseline characteristics were provided only as an incidence without an exact number of patients to protect sensitive personal information and maintain de-identified data. The primary outcome was cancer occurrence during follow-up.

### 2.2. Statistical Analysis

For the analysis, ATLAS ver. 2.7.2 was used. OHDSI analysis tools were built into the ATLAS interactive analysis platform and the OHDSI Methods Library R packages. OHDSI’s software is open-source and publicly available on the GitHub repository (https://github.com/OHDSI/ accessed on 1 September 2021). The concept sets used to define baseline characteristics and study outcomes are available at https://github.com/OHDSI/ accessed on 1 September 2021. Because OHDSI OMOP-CDM does not provide the exact number of patients for each variable, we presented baseline characteristics by incidence. We conducted propensity-score stratification and matching with more than 400 covariates according to the cohort to enhance the balance between the covariates in the two groups by minimizing the effects of potential confounding factors and selection bias. Propensity-score stratification details are presented at https://feedernet.com/project/103/analysis accessed on 1 September 2021. Propensity scores of available demographic characteristics were calculated independently in each database. The incidence of cancer was presented as cases per 1000 person-years by dividing the number of cancer cases by the total number of person years at risk and multiplying the results by 1000. We used Cox regression analysis to compare cumulative incidence based on use of RAAS inhibitors between databases. Finally, a random-effects meta-analysis was conducted to calculate the summary hazard ratio (HR) pooling effect estimates across the databases [8]. All tests were two-tailed, and *p* < 0.05 was considered statistically significant.

## 3. Results

**Figure 1:**
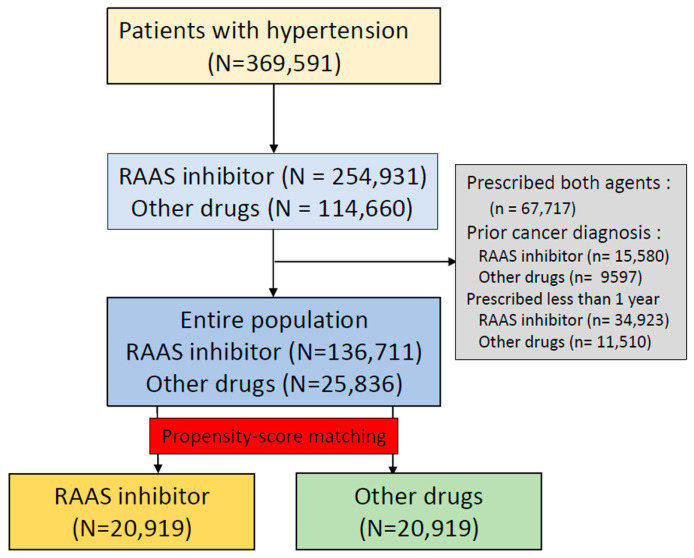
Study flowchart.

**Figure 2:**
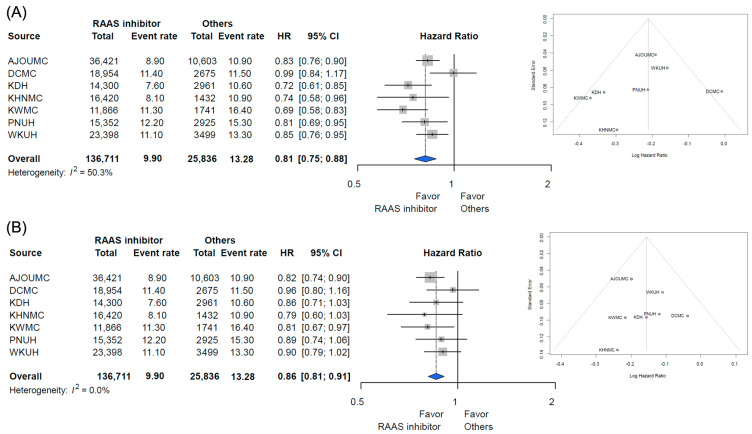
Incidence rate of all cancer in the entire cohort (**A**) adjusted for sex and age and (**B**) propensity score stratified. Abbreviations: AJOUMC, Ajou University Medical Center; DCMC, Daegu Catholic University Medical Center; KDH, Kangdong Sacred Heart Hospital; KHNMC, Kyung Hee University Hospital at Gangdong; KWMC, Kangwon Medical Center; PNUH, Pusan National University Hospital; WKUH, Wonkwang University Hospital.

**Figure 3:**
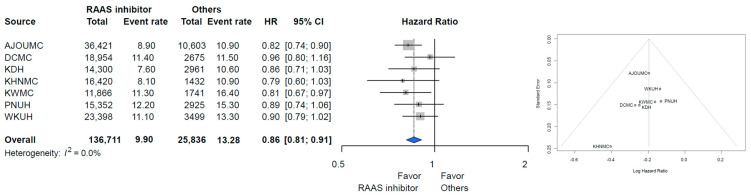
Incidence rate of all cancer in the propensity-score matched population. Abbreviations: AJOUMC, Ajou University Medical Center; DCMC, Daegu Catholic University Medical Center; KDH, Kangdong Sacred Heart Hospital; KHNMC, Kyung Hee University Hospital at Gangdong; KWMC, Kangwon Medical Center; PNUH, Pusan National University Hospital; WKUH, Wonkwang University Hospital.

A total of 369,591 patients with hypertension was identified across the seven hospitals. Among these patients, the target cohort comprised 254,931 patients who were prescribed RAAS inhibitors, and the comparator cohort was 114,660 patients using other antihypertensive drugs in the same time frame. We excluded 67,717 patients due to documented prescriptions for both RAAS inhibitors and other types of antihypertensive agents: 25,177 patients with prior cancer diagnosis and 46,433 patients with prescription for a RAAS inhibitor for less than one year. Finally, the target cohort comprised 136,711 patients with RAAS inhibitor prescription, and the comparator cohort was 25,836 patients that used other antihypertensive drugs (Figure 1). The attritions of each database are described in Figures S1–S7. The baseline characteristics of the entire cohort are shown in Table 1. Total person years were 834,543 years in the RAAS inhibitor group and 155,634 years in the other antihypertensive drugs group. The incidence rate of any cancer was 9.90 per 1000 person years in the RAAS inhibitor group and 13.28 in the other antihypertensive drugs group. The incidence rate of any cancer according to database is described in Figure 2. The meta-analysis of the whole cohort showed a lower incidence rate of any cancer in the RAAS inhibitor group (HR, 0.81; 95% confidence interval [CI], 0.75–0.88) (Figure 2). The result was similar after propensity-score stratification (HR, 0.86; 95% CI, 0.81–0.91) (Figure 2). After propensity-score matching, a total of 20,919 pairs was generated and well-balanced (Table 1). The total of person years was 125,546 years in the RAAS inhibitor group and 121,260 years in the other antihypertensive drugs group. The incidence rate of any cancer was 9.90 per 1000 person years in the RAAS inhibitor group and 13.28 in the other group. The incidence rate of any cancer in the propensity-score matched cohort is described in Figure 3. In the meta-analysis, the incidence rate of any cancer was lower in the RAAS inhibitor group (HR, 0.86; 95% CI, 0.81–0.91) (Figure 3).

We performed a separate analysis on several cancer types with a high incidence rate. Table 2 showed the incidence rate of each cancer that included present analyses in the propensity score-matched cohort. In the propensity score-matched population, the types of cancer that showed lower incidence rates by RAAS inhibitor use were liver, biliary, and gastric cancers (Figures S8–S15).

## 4. Discussion

We conducted a cohort study that used a large real-world, multicenter data set that was converted and mapped to the OMOP-CDM form of the OHDSI. The results indicated that the use of RAAS inhibitors was associated with a lower risk of cancer development compared with the use of other antihypertensive drugs. This association was valid in liver, biliary, and gastric cancers. Our findings support the reported association between the use of RAAS inhibitors and cancer development that has previously been demonstrated in numerous studies but has not been conclusive.

A growing body of evidence indicates a relationship between antihypertensive drug use and cancer [4]. For RAAS inhibitors, proto-oncogenes, oncogenes, cell signaling, microRNAs, and epigenetic factors in the RAAS system are deemed to play important roles for cancer development [6]. Indeed, the association between RAAS inhibition and cancer has been reported to range from a protective effect [9,10] to indifference [5,11], and some studies have reported that the use of RAAS inhibitors increased the risk of cancer [12,13]. The discrepancy among previous results is explained by a variety in receptors that compose the RAAS system, because these effectors and receptors of the RAAS system show an opposite action for cancer development [6]. Specifically, the two types of angiotensin II receptors are known to act counter-regulatory to each other in cancer development. Type 1 angiotensin II receptor is pro-carcinogenic, while type 2 angiotensin II receptor is anti-carcinogenic [6].

Several studies presented the increased risk of RAAS inhibitors and cancer. In the meta-analysis study about cancer occurrence, only the risk of lung cancer was significantly increased in the patients who prescribed losartan with HR of 2.41 [1]. A large Danish study that investigated skin cancers showed that ARBs, but not ACEIs, were associated with malignant melanoma [14]. On the contrary, other meta-analyses about breast cancer showed that long-term RAAS inhibitor use may have beneficial effects on breast cancer occurrence [10]. Moreover, RAAS inhibitor prescription was shown to lead to a 40 and 25% reduction in the risk of cancer recurrence and mortality [3]. Despite all of the evidence, there are conflicting results about the influence of RAAS inhibitors on cancer occurrence, recurrence, metastasis incidence, and survival.

The result of this current study showed that the use of RAAS inhibitors may be associated with GI tract cancer prevention, but not associated with other cancers that seemed to have beneficial effects, such as breast or lung cancer. This finding correlates well with current several pieces of evidence. RAAS inhibitors might reduce gastric cancer development, progression, and metastasis that is related to H. pylori infection [15]. Furthermore, blocking AT1R by losartan induces apoptotic cell death in human pancreatic cancer cells via the stimulation of the proapoptotic signaling pathways [16]. The findings of the present study may add another real-world piece of evidence to these findings.

Our results of a lower risk of cancer in the RAAS inhibitor group is in agreement with experimental studies that have demonstrated the anti-carcinogenic effects of RAAS inhibition with anti-proliferative effects in breast cancer [17], by inducing cell death in pancreatic cancer [16,18] and ameliorating liver metastases in colon cancer [19]. In addition to the current experimental studies, clinical studies and meta-analyses have demonstrated similar results [9,10]. Our study used a large dataset based on OMOP-CDM, a relatively new method for generating clinical data for investigation. We performed a propensity-score stratification and matching with a large-scale propensity-score algorithm that includes all covariates in the propensity score and produces an improved performance [20]. The primary strength of our dataset and approach is that we used real-world data based on electronic health records of seven hospitals instead of real-world datasets based on health insurance claims. While these datasets based on health insurance claims tend to lack clinical data, medical histories, and therapeutic courses, electronic health records provide more detailed and objective data [21]. Therefore, this study adds a piece of evidence to this debated issue based on a large dataset based on OMOP-CDM. Additionally, the fact that our results correspond with most of the previous meta-analyses on the same issue suggests that OMOP-CDM can be applied as a reliable tool for clinical investigation.

The following limitations should be considered when interpreting the results of this study. First, this was a retrospective study. Although we adjusted confounding factors using large-scale propensity-score stratification and matching, some of the variables were not balanced, and unmeasured factors might have affected the results. In addition, the current study was performed in Korea, so ethnic differences should be considered when interpreting our results. Second, the two main types of RAAS inhibitors were not analyzed separately. In addition, the dose or duration of RAAS inhibitor use was not provided in the dataset and the effect of specific type of RAAS inhibitors could not be analyzed, owing to low numbers. Third, our study does not provide sufficient information on whether RAAS inhibitors should be selected to prevent cancer. Despite these limitations, we provide the first real-world evidence on the association between RAAS inhibitor use and cancer occurrence using the dataset extracted from an EHR-based CDM.

## 5. Conclusions

In patients that receive antihypertensive therapy, those prescribed an RAAS inhibitor showed a lower incidence rate of cancer occurrence compared with those using other antihypertensive drugs, adding evidence to the association between RAAS inhibition and cancer occurrence.

## Figures and Tables

**Table 1:** Baseline characteristics of cohort.

				Propensity-Score Stratification			Propensity-Score Matching		
	RAAS Inhibitor (N = 136,711)	Other Drugs (N = 25,836)	SMD	RAAS Inhibitor (N = 136,711)	Other Drugs (N = 25,836)	SMD	RAAS Inhibitor (N = 20,919)	Other Drugs (N = 20,919)	SMD
Age group									
20–24	0.8	1	−0.02	0.7	0.9	−0.02	0.7	0.9	−0.02
25–29	0.8	1.7	−0.08	1.3	1.3	0.01	1.3	1.2	0.01
30–34	1.5	2.4	−0.07	1.8	1.9	−0.01	1.7	1.8	−0.01
35–39	3	3.7	−0.04	3.2	3.5	−0.02	3.2	3.4	−0.02
45–49	7.8	6.9	0.03	7.5	7.6	0	7.3	7.4	0
50–54	10.7	9.2	0.05	10.4	10.4	0	10.3	10.3	0
55–59	12.4	11.1	0.04	12.6	12.3	0.01	12.6	12.3	0.01
60–64	13.2	11.9	0.04	13.4	13.3	0	13.4	13.4	0
65–69	13.1	12.3	0.02	13.1	13.1	0	13.2	13.1	0
70–74	12.4	12.6	−0.01	12.8	12.4	0.01	12.9	12.5	0.01
80–84	5.9	7	−0.05	5.4	5.5	0	5.5	5.6	0
85–89	2.4	3.1	−0.04	2.1	2.2	0	2.2	2.3	0
Gender: female	45.3	53.5	−0.16	56.6	55.1	0.03	56.7	55.2	0.03
Medical history: General									
Acute respiratory disease	2	3.2	−0.08	2.8	2.5	0.02	2.8	2.5	0.02
Diabetes mellitus	17.6	10.7	0.2	9.5	9.9	−0.01	9.3	9.8	−0.01
Hyperlipidemia	14.3	7.2	0.23	9.1	9.4	−0.01	9.2	9.5	−0.01
Hypertensive disorder	50.1	37.6	0.25	42.8	43.1	−0.01	42.4	42.7	−0.01
Osteoarthritis	1.5	1.7	−0.02	2.3	2.1	0.01	2.3	2.1	0.01
Pneumonia	2.9	6	−0.15	3.5	3.4	0.01	3.5	3.4	0.01
Renal impairment	5.2	5.7	−0.02	4.3	4.1	0.01	4.3	4.1	0.01
Rheumatoid arthritis	0.4	1.9	−0.14	1.8	1.5	0.02	1.7	1.4	0.02
Visual system disorder	8.2	7.6	0.02	8.4	8.6	−0.01	8.5	8.7	−0.01
Medical history: Cardiovascular disease									
Cerebrovascular disease	5.9	4.1	0.08	5	4.9	0	5	5	0
Coronary arteriosclerosis	5.7	1.4	0.24	2.2	2.1	0.01	2.1	2	0.01
Heart disease	34.4	11.6	0.56	14.4	14.8	−0.01	14.5	14.9	−0.01
Ischemic heart disease	18.8	4.7	0.45	7.2	7.1	0	7.1	7.1	0
Venous thrombosis	0.4	0.8	−0.05	0.5	0.5	0.01	0.5	0.5	0.01

Values are percentage. SMD: Standardized mean difference.

**Table 2:** Hazard ratio of each cancer in the overall and propensity score-matched cohort.

	RAAS Inhibtor	Others	Unadjusted HR (95% CI)	PS Matched Pairs	PS Adjusted HR (95% CI)
Breast cancer	66,701	16,930	1.02 (0.82–1.26)	13,157	1.07 (0.71–1.63)
Colon cancer	146,043	30,543	0.82 (0.67–1.00)	24,216	0.80 (0.59–1.08)
Gastric cancer	145,997	30,456	0.79 (0.68–0.92)	24,170	0.79 (0.62–1.00)
Liver cancer	146,854	30,367	0.53 (0.46–0.60)	24,393	0.71 (0.53–0.96)
Lung cancer	146,882	30,962	0.68 (0.52–0.90)	24,496	0.80 (0.55–1.16)
Gynecological cancer	67,139	16,993	0.79 (0.51–1.24)	13,230	1.48 (0.91–2.40)
Pancreaticobiliary cancer	147,049	31,015	0.62 (0.54–0.72)	24,565	0.61 (0.45–0.82)
Prostate cancer	78,920	13,704	0.77 (0.58–1.02)	10,838	1.00 (0.64–1.55)

RAAS, renin angiotensin aldosterone system; HR, hazard ration; CI, confidence interval; PS, propensity score.

## Data Availability

The data are not publicly available due to our institutional guidelines.

## Supplementary Materials

The following supporting information can be downloaded at: https://www.mdpi.com/article/10.3390/diagnostics12020263/s1, Figure S1: Balance between the groups before and after propensity score matching of AJOUMC cohort for all cancer occurrence. Figure S2: Balance between the groups before and after propensity score matching of DCMC cohort for all cancer occurrence. Figure S3: Balance between the groups before and after propensity score matching of KDH cohort for all cancer occurrence. Figure S4: Balance between the groups before and after propensity score matching of KHNMC cohort for all cancer occurrence. Figure S5: Balance between the groups before and after propensity score matching of KWMC cohort for all cancer occurrence. Figure S6: Balance between the groups before and after propensity score matching of PNUH cohort for all cancer occurrence. Figure S7: Balance between the groups before and after propensity score matching of WKUH cohort for all cancer occurrence. Figure S8: Incidence rate of breast cancer in the entire cohort (A) adjusted for sex and age, (B) propensity score stratified and (C) propensity-score matched population. Figure S9: Incidence rate of colon cancer in the entire cohort (A) adjusted for sex and age, (B) propensity score stratified and (C) propensity-score matched population. Figure S10: Incidence rate of gastric cancer in the entire cohort (A) adjusted for sex and age, (B) propensity score stratified and (C) propensity-score matched population. Figure S11: Incidence rate of liver cancer in the entire cohort (A) adjusted for sex and age, (B) propensity score stratified and (C) propensity-score matched population. Figure S12: Incidence rate of lung cancer in the entire cohort (A) adjusted for sex and age, (B) propensity score stratified and (C) propensity-score matched population. Figure S13: Incidence rate of gynecological cancer in the entire cohort (A) adjusted for sex and age, (B) propensity score stratified and (C) propensity-score matched population. Figure S14: Incidence rate of pancreaticobiliary cancer in the entire cohort (A) adjusted for sex and age, (B) propensity score stratified and (C) propensity-score matched population. Figure S15: Incidence rate of prostate cancer in the entire cohort (A) adjusted for sex and age, (B) propensity score stratified and (C) propensity-score matched population.
