# Supplementary material for: Renin-Angiotensin-Aldosterone System Inhibitors and Risk of Cancer: A Population-Based Cohort Study Using a Common Data Model
Source: Diagnostics (Basel). 2022 Jan 21;12(2):263. doi: 10.3390/diagnostics12020263 (PMC8871518; doi:10.3390/diagnostics12020263)
Supplement: Supplementary file 1 [file diagnostics-12-00263-s001.zip › diagnostics-1512699-supplementary.pdf]

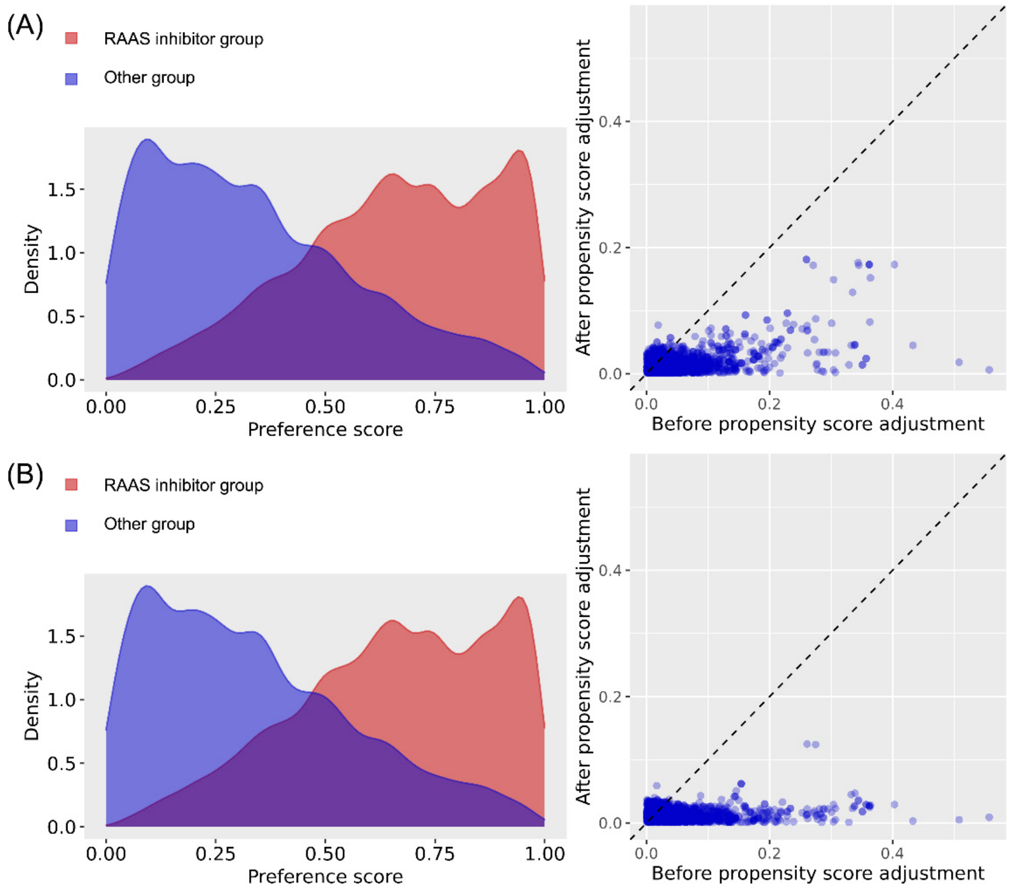

Figure S1. Balance between the groups before and after propensity score matching of AJOUMC cohort for all cancer occurrence.

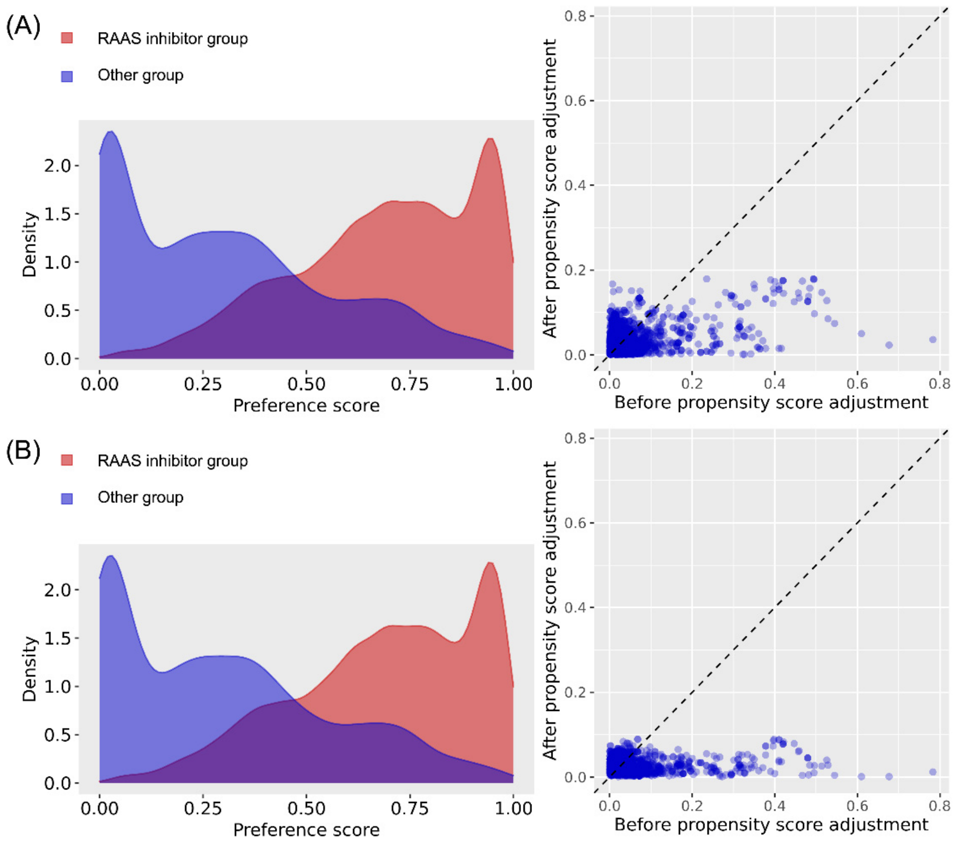

Figure S2. Balance between the groups before and after propensity score matching of DCMC cohort for all cancer occurrence.

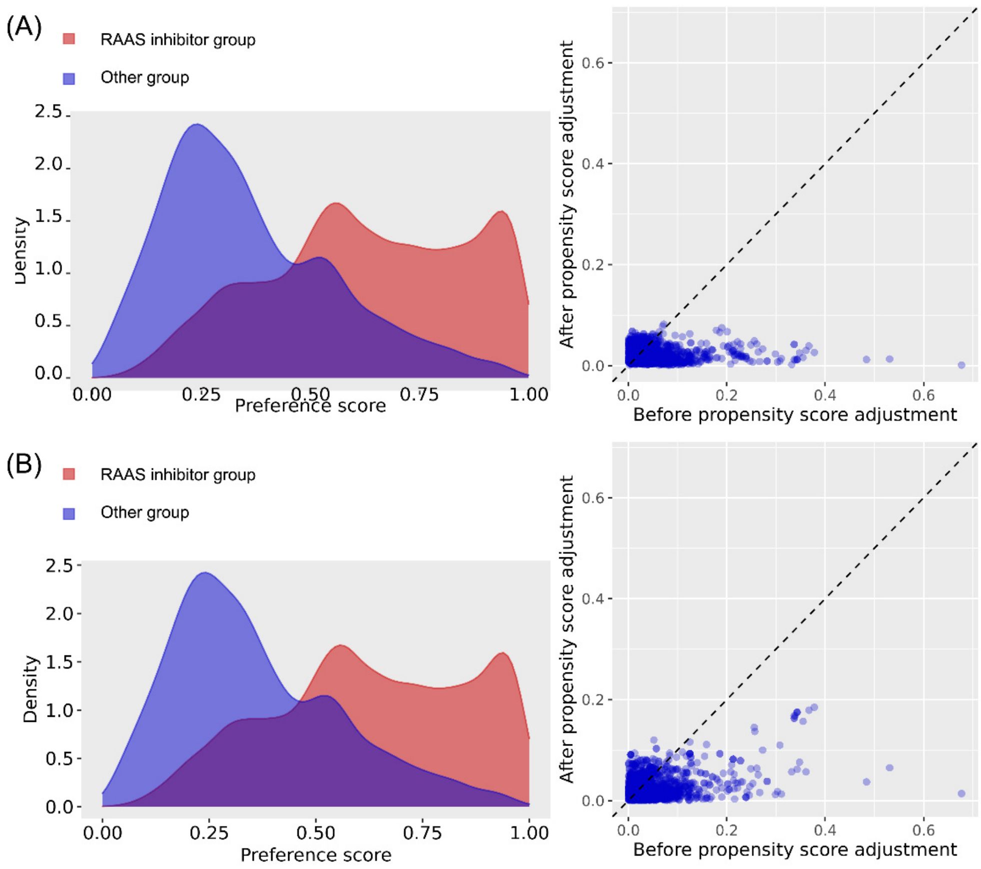

Figure S3. Balance between the groups before and after propensity score matching of KDH cohort for all cancer occurrence.

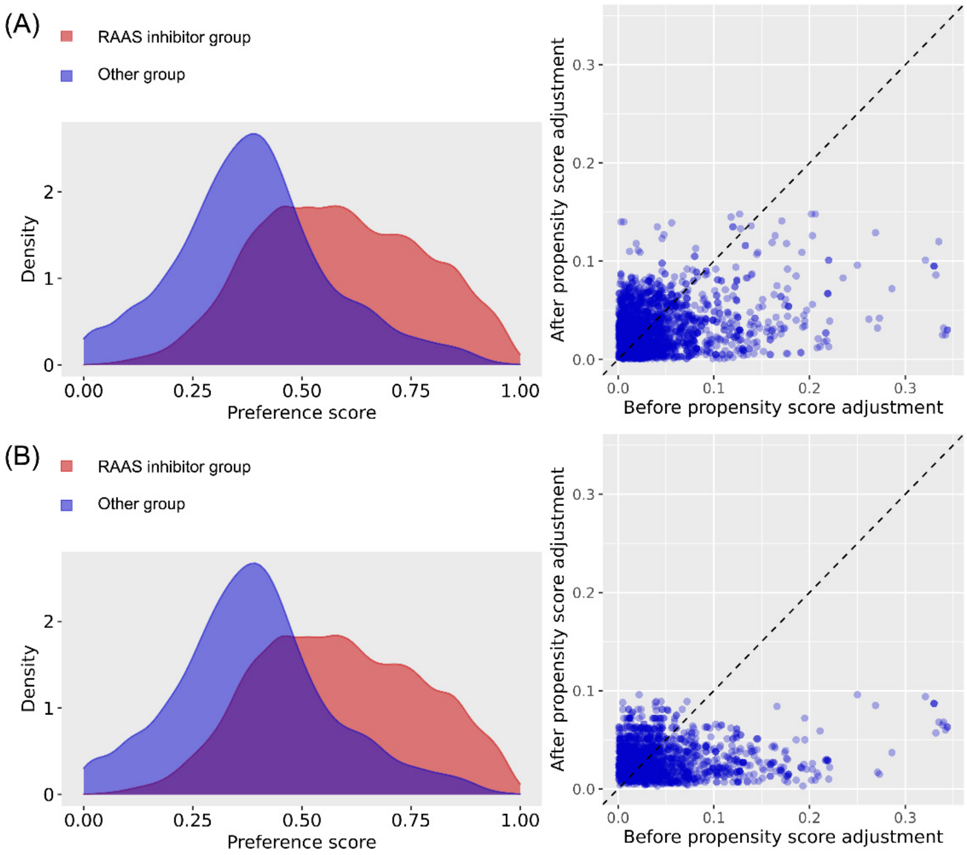

Figure S4. Balance between the groups before and after propensity score matching of KHNMC cohort for all cancer occurrence.

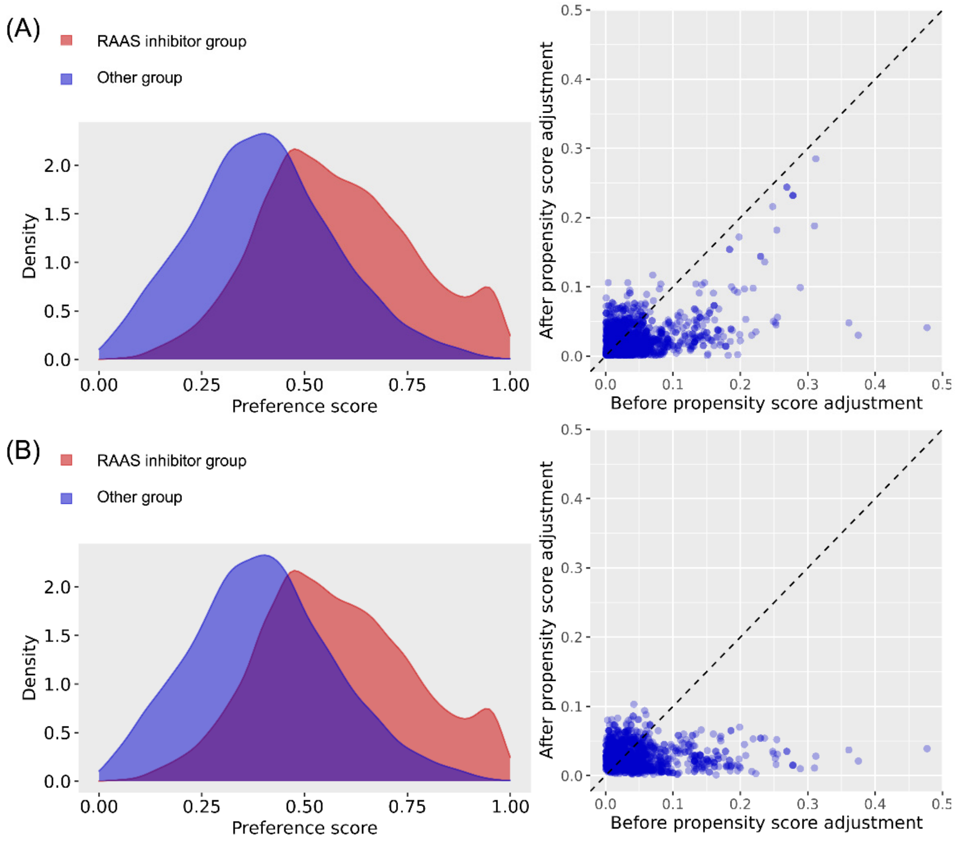

Figure S5. Balance between the groups before and after propensity score matching of KWMC cohort for all cancer occurrence.

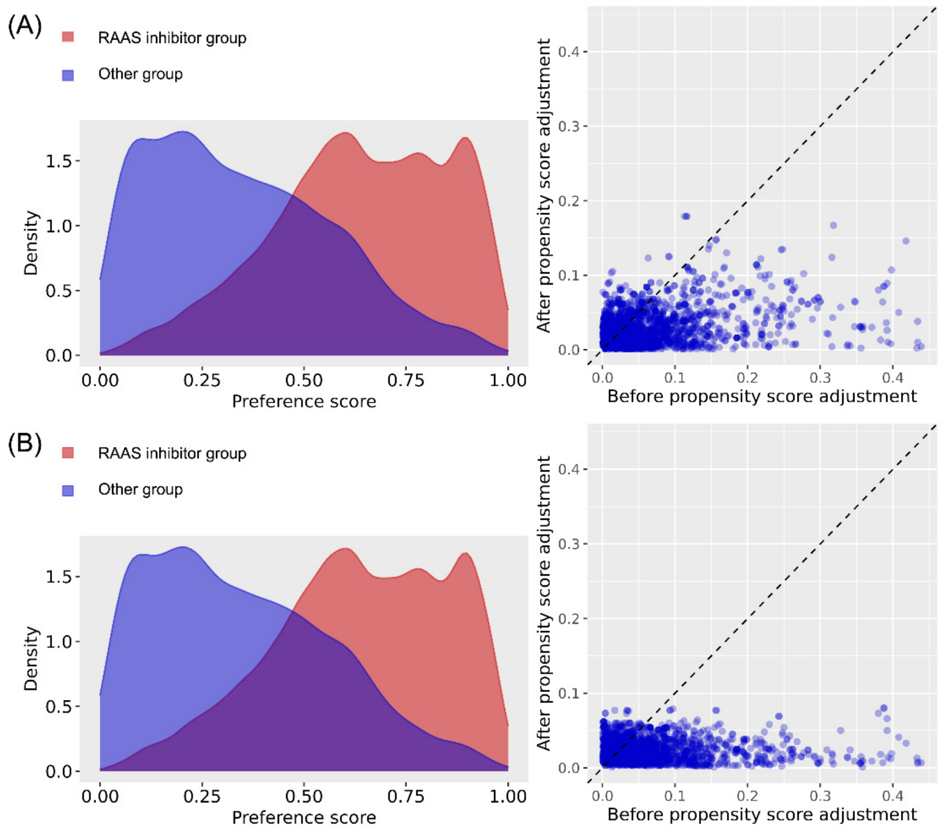

Figure S6. Balance between the groups before and after propensity score matching of PNUH cohort for all cancer occurrence.

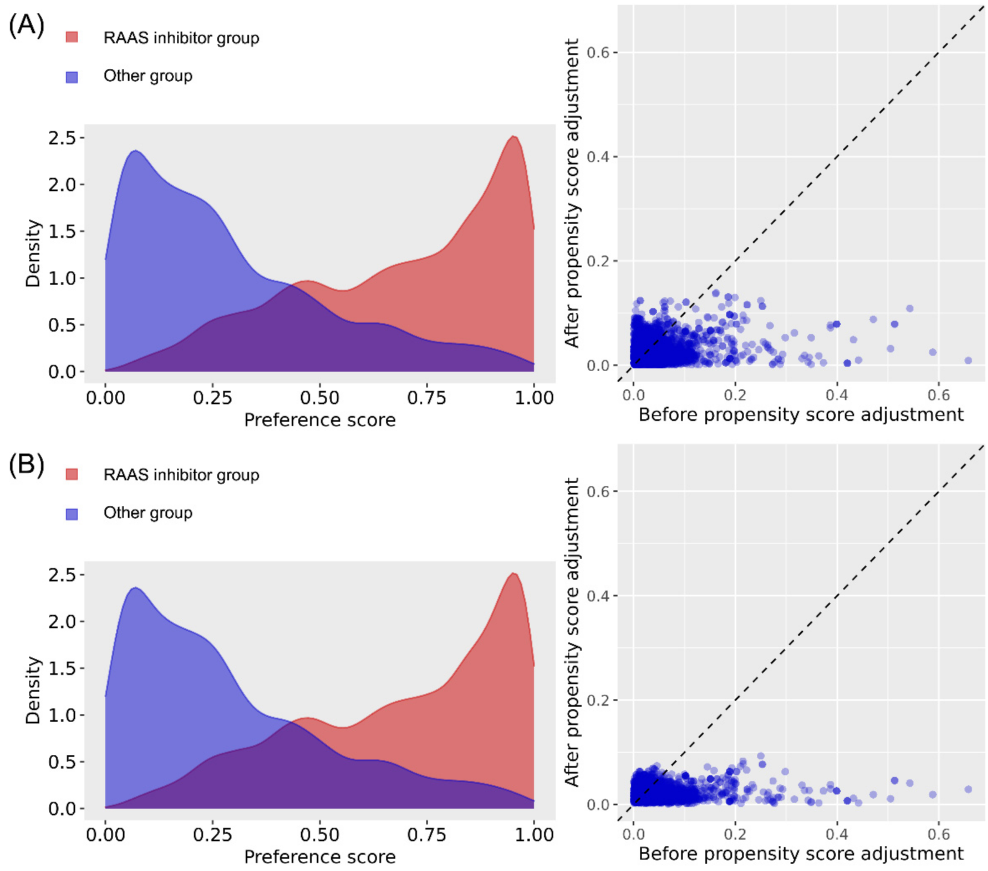

Figure S7. Balance between the groups before and after propensity score matching of WKUH cohort for all cancer occurrence.

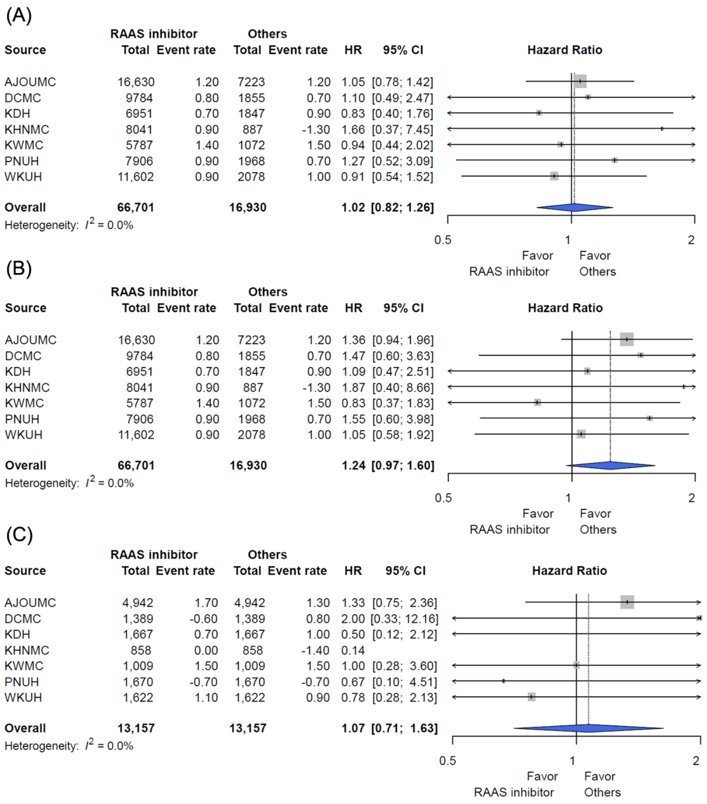

(A)

Source	RAAS inhibitor Total	RAAS inhibitor Event rate	Others Total	Others Event rate	HR	95% CI
AJOUMC	16,630	1.20	7223	1.20	1.05	[0.78; 1.42]
DCMC	9784	0.80	1855	0.70	1.10	[0.49; 2.47]
KDH	6951	0.70	1847	0.90	0.83	[0.40; 1.76]
KHNMC	8041	0.90	887	-1.30	1.66	[0.37; 7.45]
KWMC	5787	1.40	1072	1.50	0.94	[0.44; 2.02]
PNUH	7906	0.90	1968	0.70	1.27	[0.52; 3.09]
WKUH	11,602	0.90	2078	1.00	0.91	[0.54; 1.52]
**Overall**	**66,701**		**16,930**		**1.02**	**[0.82; 1.26]**

Heterogeneity: $I^2 = 0.0\%$

(B)

Source	RAAS inhibitor Total	RAAS inhibitor Event rate	Others Total	Others Event rate	HR	95% CI
AJOUMC	16,630	1.20	7223	1.20	1.36	[0.94; 1.96]
DCMC	9784	0.80	1855	0.70	1.47	[0.60; 3.63]
KDH	6951	0.70	1847	0.90	1.09	[0.47; 2.51]
KHNMC	8041	0.90	887	-1.30	1.87	[0.40; 8.66]
KWMC	5787	1.40	1072	1.50	0.83	[0.37; 1.83]
PNUH	7906	0.90	1968	0.70	1.55	[0.60; 3.98]
WKUH	11,602	0.90	2078	1.00	1.05	[0.58; 1.92]
**Overall**	**66,701**		**16,930**		**1.24**	**[0.97; 1.60]**

Heterogeneity: $I^2 = 0.0\%$

(C)

Source	RAAS inhibitor Total	RAAS inhibitor Event rate	Others Total	Others Event rate	HR	95% CI
AJOUMC	4,942	1.70	4,942	1.30	1.33	[0.75; 2.36]
DCMC	1,389	-0.60	1,389	0.80	2.00	[0.33; 12.16]
KDH	1,667	0.70	1,667	1.00	0.50	[0.12; 2.12]
KHNMC	858	0.00	858	-1.40	0.14	
KWMC	1,009	1.50	1,009	1.50	1.00	[0.28; 3.60]
PNUH	1,670	-0.70	1,670	-0.70	0.67	[0.10; 4.51]
WKUH	1,622	1.10	1,622	0.90	0.78	[0.28; 2.13]
**Overall**	**13,157**		**13,157**		**1.07**	**[0.71; 1.63]**

Heterogeneity: $I^2 = 0.0\%$

Figure S8. Incidence rate of breast cancer in the entire cohort (A) adjusted for sex and age, (B) propensity score stratified and (C) propensity-score matched population.

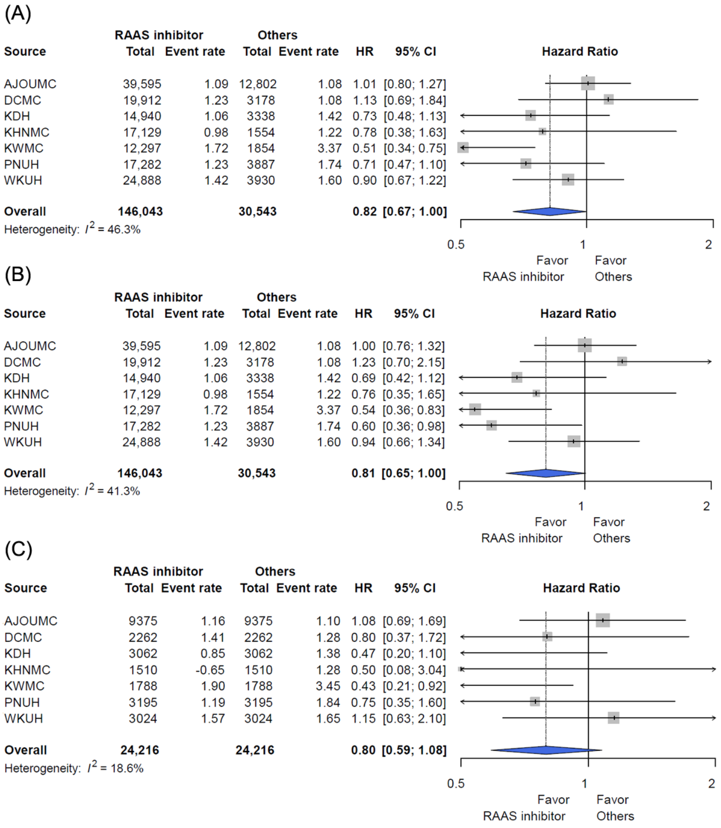

(A)

Source	RAAS inhibitor Total	RAAS inhibitor Event rate	Others Total	Others Event rate	HR	95% CI
AJOUMC	39,595	1.09	12,802	1.08	1.01	[0.80; 1.27]
DCMC	19,912	1.23	3178	1.08	1.13	[0.69; 1.84]
KDH	14,940	1.06	3338	1.42	0.73	[0.48; 1.13]
KHNMC	17,129	0.98	1554	1.22	0.78	[0.38; 1.63]
KWMC	12,297	1.72	1854	3.37	0.51	[0.34; 0.75]
PNUH	17,282	1.23	3887	1.74	0.71	[0.47; 1.10]
WKUH	24,888	1.42	3930	1.60	0.90	[0.67; 1.22]
**Overall**	**146,043**		**30,543**		**0.82**	**[0.67; 1.00]**

Heterogeneity: $I^2 = 46.3\%$

(B)

Source	RAAS inhibitor Total	RAAS inhibitor Event rate	Others Total	Others Event rate	HR	95% CI
AJOUMC	39,595	1.09	12,802	1.08	1.00	[0.76; 1.32]
DCMC	19,912	1.23	3178	1.08	1.23	[0.70; 2.15]
KDH	14,940	1.06	3338	1.42	0.69	[0.42; 1.12]
KHNMC	17,129	0.98	1554	1.22	0.76	[0.35; 1.65]
KWMC	12,297	1.72	1854	3.37	0.54	[0.36; 0.83]
PNUH	17,282	1.23	3887	1.74	0.60	[0.36; 0.98]
WKUH	24,888	1.42	3930	1.60	0.94	[0.66; 1.34]
**Overall**	**146,043**		**30,543**		**0.81**	**[0.65; 1.00]**

Heterogeneity: $I^2 = 41.3\%$

(C)

Source	RAAS inhibitor Total	RAAS inhibitor Event rate	Others Total	Others Event rate	HR	95% CI
AJOUMC	9375	1.16	9375	1.10	1.08	[0.69; 1.69]
DCMC	2262	1.41	2262	1.28	0.80	[0.37; 1.72]
KDH	3062	0.85	3062	1.38	0.47	[0.20; 1.10]
KHNMC	1510	-0.65	1510	1.28	0.50	[0.08; 3.04]
KWMC	1788	1.90	1788	3.45	0.43	[0.21; 0.92]
PNUH	3195	1.19	3195	1.84	0.75	[0.35; 1.60]
WKUH	3024	1.57	3024	1.65	1.15	[0.63; 2.10]
**Overall**	**24,216**		**24,216**		**0.80**	**[0.59; 1.08]**

Heterogeneity: $I^2 = 18.6\%$

Figure S9. Incidence rate of colon cancer in the entire cohort (A) adjusted for sex and age, (B) propensity score stratified and (C) propensity-score matched population.

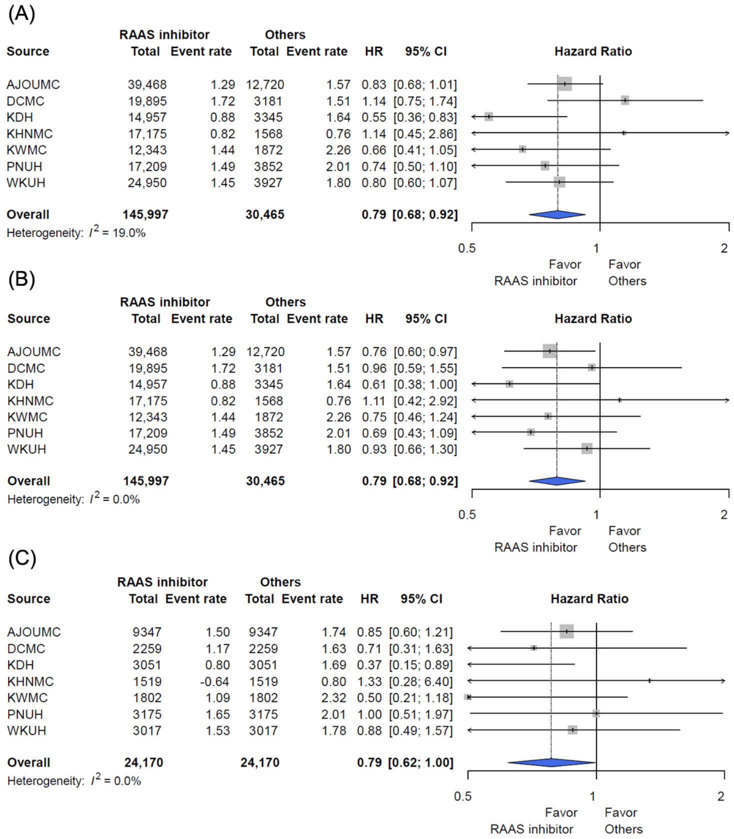

Figure S10. Incidence rate of gastric cancer in the entire cohort (A) adjusted for sex and age, (B) propensity score stratified and (C) propensity-score matched population.

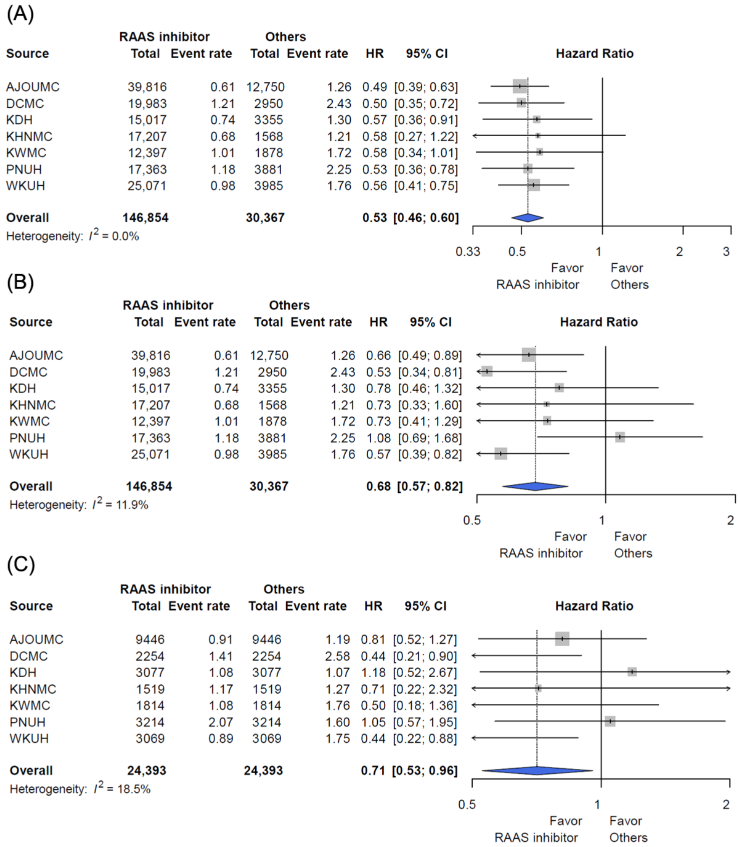

Figure S11. Incidence rate of liver cancer in the entire cohort (A) adjusted for sex and age, (B) propensity score stratified and (C) propensity-score matched population.

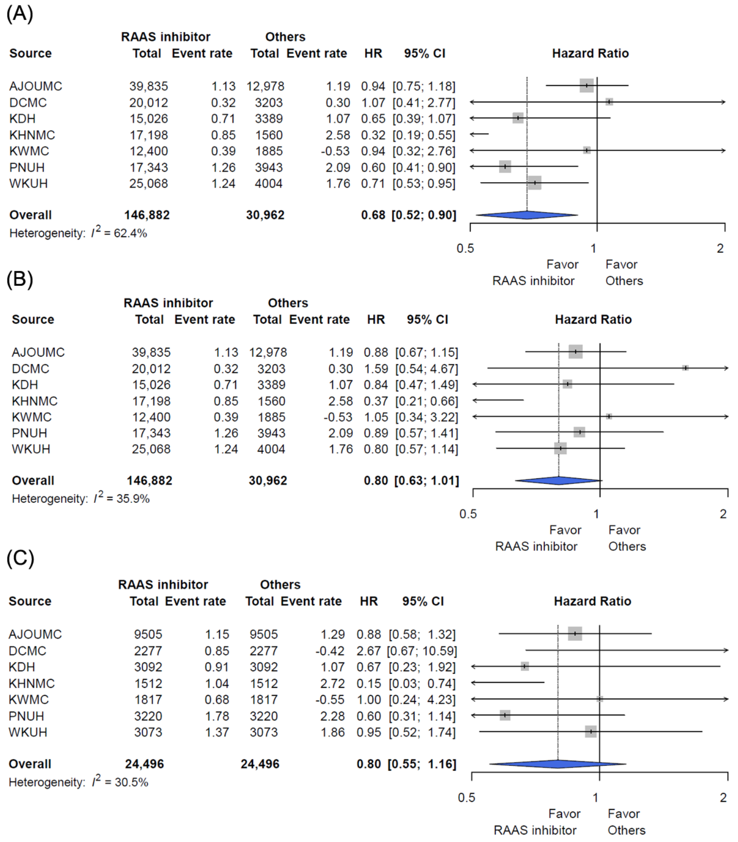

Figure S12. Incidence rate of lung cancer in the entire cohort (A) adjusted for sex and age, (B) propensity score stratified and (C) propensity-score matched population.

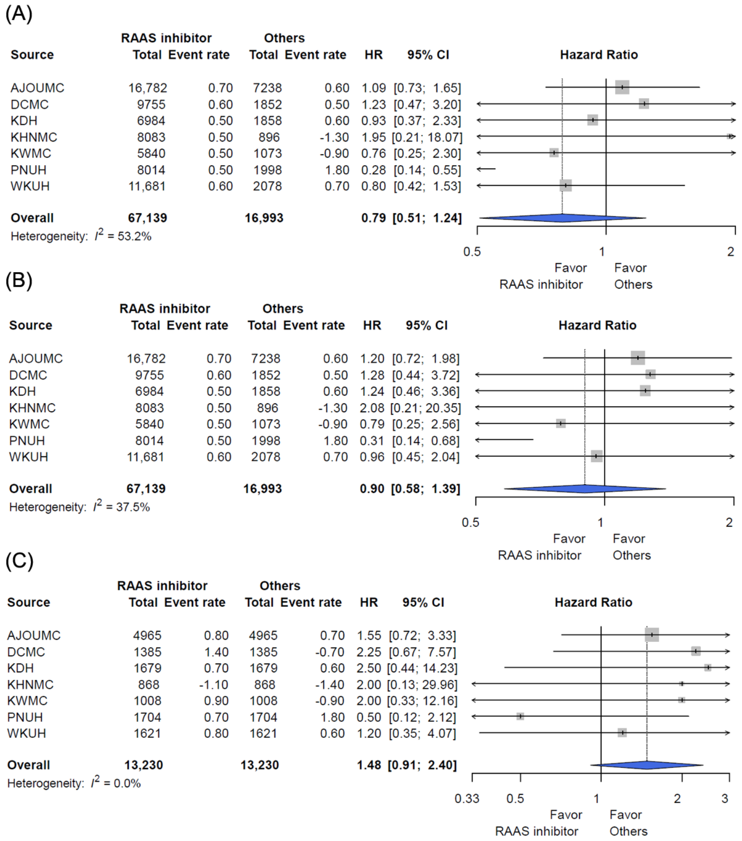

Figure S13. Incidence rate of gynecological cancer in the entire cohort (A) adjusted for sex and age, (B) propensity score stratified and (C) propensity-score matched population.

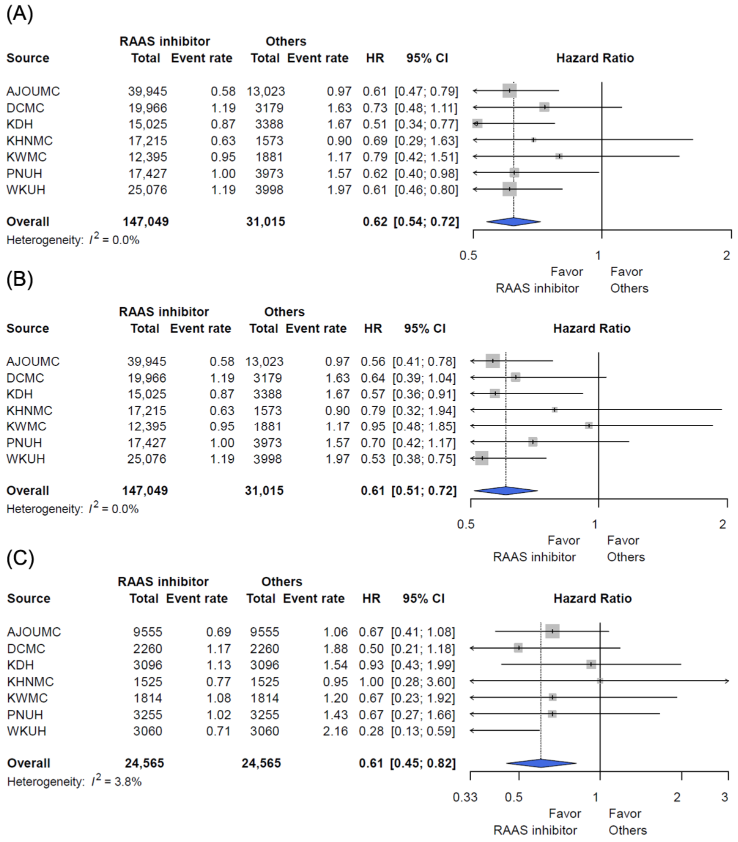

Figure S14. Incidence rate of pancreaticobiliary cancer in the entire cohort (A) adjusted for sex and age, (B) propensity score stratified and (C) propensity-score matched population.

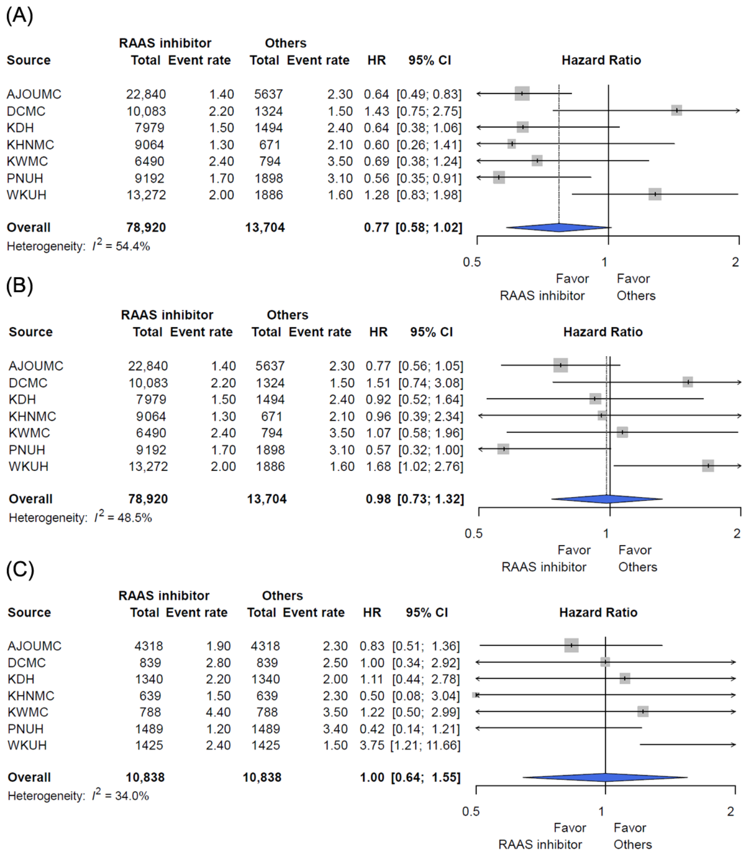

Figure S15. Incidence rate of prostate cancer in the entire cohort (A) adjusted for sex and age, (B) propensity score stratified and (C) propensity-score matched population.
